# A Contemporary Narrative Review of Sodium Homeostasis Mechanisms, Dysnatraemia, and the Clinical Relevance in Adult Critical Illness

**DOI:** 10.3390/jcm14196914

**Published:** 2025-09-29

**Authors:** Vignesh Raman, Mahesh Ramanan, Felicity Edwards, Kevin B. Laupland

**Affiliations:** 1 Faculty of Health, Queensland University of Technology, Brisbane, QLD 4006, Australia; vignesh.raman@health.qld.gov.au (V.R.); mahesh.ramanan@health.qld.gov.au (M.R.); felicity.edwards@qut.edu.au (F.E.); 2 Department of Neurosurgery, Royal Brisbane and Women’s Hospital, Brisbane, QLD 4006, Australia; 3 Department of Intensive Care Services, Royal Brisbane and Women’s Hospital, Brisbane, QLD 4006, Australia; 4 Critical Care Division, The George Institute for Global Health, University of New South Wales, Sydney, NSW 2052, Australia

**Keywords:** sodium, dysnatraemia, hypertonic saline, critical illness

## Abstract

Amongst critically ill patients managed in the intensive care unit (ICU) setting, disorders of sodium and water balance, or dysnatraemias, are commonly encountered either at time of admission or during ICU stay. There is extensive literature associating both extremities of incident dysnatraemia, hyponatraemia, and hypernatremia, with higher mortality and morbidity amongst a range of ICU disease populations. Therefore, a comprehensive understanding of sodium homeostasis mechanisms, effects of deranged sodium physiology, comprehensive diagnostic workup, and avoidance of suboptimal management are paramount to the critical care clinician. This narrative review incorporated a PubMed search to summarise contemporary literature perspectives of (a) sodium homeostasis mechanisms, (b) descriptions of dysnatraemia, (c) ICU-specific challenges to dysnatraemia diagnosis, (d) associated clinical outcomes in critically ill populations, and (e) ongoing paucity to deranged sodium physiology literature relevant to daily ICU practice. The purpose of this review is to both guide critical care clinicians towards performing a diagnostic approach to dysnatraemia with integrated physiology for a tailored treatment strategy as well as highlight ongoing research priorities in the realm of deranged sodium physiology.

## 1. Introduction

Disorders of serum sodium and water balance, collectively called dysnatraemia, are the commonest electrolyte disorders encountered amongst critically ill patients managed in the intensive care unit (ICU) setting [1]. Both extremes of dysnatraemia, hyponatraemia and hypernatraemia, have previously been associated with higher mortality and morbidity outcomes in medical, surgical, and neurological ICU patient populations [2,3,4,5,6,7,8,9]. Consequently, a comprehensive understanding of sodium homeostasis mechanisms, as well as diagnostic and management paradigms for dysnatraemia, are paramount to the critical care clinician. There are several nuanced differences to dysnatraemia in the ICU population ranging from primary disease severity, the presence of multiple disease processes, difficulties in measuring fluid balance, available treatment, and monitoring modalities not available to the general hospital population. Further, studying the epidemiology of ICU dysnatraemia is challenged by varied definitions based on serum sodium cut-offs, population heterogeneity, local diagnostic practices, and resource availabilities. There is also a growing body of literature supporting greater magnitudes of dynamic change in serum sodium amongst specific ICU disease populations being associated with increased mortality and morbidity, independent of incident dysnatraemia [7,9,10,11]. However, the limited literature is underpowered to confirm whether this is causal relationship, or secondary to illness severity and treatment response. Further, it remains unclear whether targeting a certain serum sodium concentration value is associated with improved clinical outcomes; this would be particularly invaluable to establish in certain disease categories encountered in the ICU such as traumatic brain injury (TBI) and spontaneous subarachnoid haemorrhage (SAH), where a focus on serum sodium concentration is considered basic ICU care [12].

This contemporary narrative review summarises five key aspects of the current dysnatraemia literature including (a) current understandings of sodium homeostasis mechanisms, (b) descriptions of dysnatraemia, (c) ICU-specific challenges to dysnatraemia diagnosis and management, (d) associated clinical outcomes in critically ill populations, and (e) ongoing paucity of the literature on deranged sodium physiology relevant to daily ICU practice. This review aims to both guide critical care clinicians towards working up dysnatraemia in the ICU setting with a tailored treatment strategy and to highlight ongoing research priorities in the realm of deranged sodium physiology.

## 2. Methods

PubMed^®^ internet database was searched for review content. The terms were used as follows: ‘sodium’, ‘dysnatraemia’ ‘hyponatraemia’, ‘hypernatraemia’, ‘pathophysiology’, ‘diagnosis’, and ‘management’ (May 2025). Inclusion criteria: articles in English; articles about serum sodium regulation, hyponatraemia and hypernatraemia diagnosis and management in the ICU, research with adult patients, meta-analyses, systematic reviews, clinical studies, including randomised controlled trials, observational studies, historical data, and systematic reviews. Exclusion criteria: articles in a language other than English, animal studies, research with child and adolescent (age < 18 years old), studies in general hospital (non-ICU) patients, case studies, and narrative reviews. Grey literature was also included. Included articles were published between 1990 and 2025.

## 3. Sodium Haemostasis

### 3.1. Osmoregulation

To maintain homeostasis, cell size, and function, the physiological regulation of extracellular fluid (ECF) osmolality, or osmoregulation, is paramount [13]. Normal ECF osmolality is between 275 and 285 mOsm/L and sodium, the predominant osmolyte cation in ECF, has a normal concentration between 135 and 145 mmol/L and is the most important determinant of this [13,14]. Thus, osmoregulation of ECF and the regulation of sodium concentration are closely intertwined [15]. Cell membranes contain water-permeable aquaporin channels, and water diffuses freely between the ECF and intracellular space, implying a near identical osmolality in these two compartments [13]. High or low extracellular sodium concentrations can lead to osmotic water shifts that cause cellular shrinkage or swelling, respectively, both of which cause subsequent cellular dysfunction [15].

**Figure 1:**
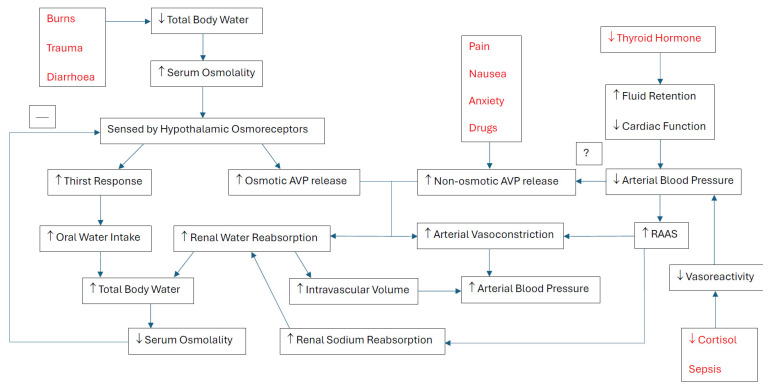
Regulatory mechanisms for sodium and water with downstream effects on serum osmolality and arterial blood pressure. AVP = arginine vasopressin, RAAS = renin–angiotensin–aldosterone system, — = negative feedback, ? = proposed mechanism.

Osmoregulation of ECF and regulation of sodium concentration are determined primarily by two mechanisms functioning in parallel: (1) the osmoreceptor–arginine vasopressin (AVP) response and (2) the thirst response, both of which involve the central nervous system (CNS) in the afferent limb (see Figure 1) [15,16,17]. The osmoreceptor–AVP response involves osmoreceptor cells of the anterior hypothalamus that shrink in response to increased serum osmolarity (predominantly driven by increased serum sodium), leading to subsequent signalling to the supraoptic nuclei and release of stored arginine vasopressin (AVP) from the posterior pituitary [15]. AVP released into the bloodstream stimulates V2 receptors on renal collecting tubules, leading to activation of adenylate cyclase and cyclic adenosine monophosphate, upregulating the number of aquaporin-2 water channels in the apical membrane of the renal collecting tubules, leading to increased water reabsorption [18,19]. Secretion of AVP in response to an osmotic stimulus is rapid, so serum AVP levels can increase several-fold within minutes, providing a rapid means for altering renal excretion of water [15].

The thirst centre is located in the preoptic nucleus of the hypothalamus as well as the anteroventral wall of the third ventricle that promotes AVP release, and stimulation here results in an immediate drive to drink [15]. Stimulants for the thirst response are essentially causes of intracellular dehydration of the thirst centre and includes primarily an increased serum osmolarity, but also non-osmotic triggers such as reduced arterial effective blood volume (e.g., hypovolaemia, congestive cardiac failure, or sepsis), increased angiotensin II and dry mouth or oesophageal mucous membranes [15,17]. Gastrointestinal distention may partially alleviate the thirst response, but this mechanism is short-lived and the desire to drink is only quenched once serum osmolarity or circulating blood volume returns to baseline [20,21].

The body is continuously challenged by dehydration and trends towards increasing ECF osmolarity from the kidneys, constantly attempting to excrete water-soluble metabolites, drugs and toxins, along with water lost through the lung, gastrointestinal tract, and sweat [16,21]. When intact, the thirst response threshold for drinking is as much as serum sodium increase of 2 mmol/L above a person’s baseline [17]. Contrarily, sodium salt consumption as much as six times normal result in minimal disturbances to serum sodium concentrations when the osmotic–AVP response and thirst response remain functional [15].

### 3.2. Non-Osmotic–AVP Release

A non-osmotic trigger for AVP is reduced arterial effective blood volume, where AVP released from the posterior pituitary gland again stimulates increased renal water reabsorption, but also acts on V1a receptors on vascular smooth muscle, leading to arterial vasoconstriction and increase in total peripheral resistance [18,19,22]. Two other compensatory mechanisms activated in response to reduced arterial effective blood volume are the (1) renin–angiotensin–aldosterone system (RAAS) and (2) sympathetic system [18,19]. The increase in sympathetic tone stimulates the RAAS through renal–adrenergic stimulation and by an increase in sympathetic tone and RAAS to increase systemic vascular resistance compensating for reduced arterial effective blood volume [18,19]. Although these mechanisms allow for maintaining end-organ perfusion in the acute setting, prolonged, inappropriate activation in the setting of congestive cardiac failure and cirrhosis can lead to sodium and water retention, manifesting as pulmonary oedema and ascites [18,19]. Another compensatory mechanism of note is mediated by ‘low-pressure’ receptors located in cardiac atria, which in response to an increase in transmural atrial pressure, inhibit the release of pituitary AVP, decrease renal vascular resistance, and result in increased water and sodium excretion [18]. In the setting of cardiac strain, cardiac atrial stretch also results in cardiomyocyte secretion of atrial natriuretic peptide (ANP) (C-terminal and N-terminal types) and B-type natriuretic peptide (BNP), promoting natriuresis (urine sodium excretion), secondary free water diuresis, and subsequent intravascular volume reduction [18]. There is also speculation that BNP is also released from the thalamus in response to physiological stress and severe brain injury [12,23,24,25].

Other triggers for non-osmotic–AVP release resulting in water retention include sympathetic stimulation (e.g., pain, nausea, stress, and anxiety) or certain drugs [13,15]. Nicotine, morphine and cyclophosphamide can stimulate AVP release, whereas alcohol inhibits AVP release and partly explains marked diuresis observed with alcohol consumption [15]. In cases of nausea, there have been 100-fold increases observed in serum AVP compared with baseline [15].

### 3.3. Angiotensin II and Aldosterone

Angiotensin II, a modified peptide hormone initially synthesised by the liver, and aldosterone, an endogenous mineralocorticoid steroid hormone from the adrenal cortex, play a pivotal role in regulating renal tubular sodium reabsorption, but only regulate serum sodium concentration under extreme conditions [15]. Both angiotensin II and aldosterone increase the quantity of ECF sodium, but also increase the ECF volume by increasing reabsorption of water [15]. Also, if the osmotic–AVP and thirst response mechanisms are functional, any tendency toward increased serum sodium concentration is compensated for by increased serum AVP secretion and kidney-mediated water retention or increased water intake, both of which will lead to ECF dilution back toward normal [14,17]. Even amongst patients with primary hyperaldosteronism with extremely high levels of aldosterone, the serum sodium concentration usually increases only approximately 3–5 mmol/L above normal [15].

In the case of complete loss of aldosterone secretion (e.g., post-adrenalectomy or Addison’s disease), there is significant renal loss of sodium, which can lead to a marked reduction in serum sodium concentration [14,17]. This is partly due to large losses of sodium eventually causing severe ECF volume depletion and decreased blood pressure, which can activate the thirst response [14,17]. This activation leads to subsequent dilution of the serum sodium concentration, even though the increased water intake helps minimise the decrease in body fluid volumes under these conditions [15]. Thus, there are extreme situations in which hyponatraemia can occur, even with functional osmotic–AVP and thirst response mechanisms.

### 3.4. Cortisol and Thyroid Function

Cortisol, an endogenous glucocorticoid steroid hormone released from the adrenal cortex, results from stimulation of pituitary-released adrenocorticotropic hormone and also has a minor role in sodium reabsorption from the renal distal tubules and collecting [26]. Adrenal insufficiency is seen often in acute brain injury, resulting in a significant reduction in aldosterone and cortisol, consequently impacting on vascular tone, intravascular volume, and sodium retention [27,28]. This is because adrenal insufficiency with glucocorticoid deficiency results in decreased effective circulating volume and non-osmotic increased AVP and thirst response [13].

There is no clear mechanistic association between thyroid hormones and sodium or water balance regulation to explain the observed association between hypothyroidism and low serum sodium concentrations [13]. Some have proposed that fluid retention, impaired cardiac and renal function, may lead to decreased effective circulating volume and non-osmotic increased AVP and thirst response [29].

## 4. Dysnatraemia

### 4.1. Categories, Causes, and ICU Epidemiology

Dysnatraemia, the overarching term for serum sodium concentration outside the normal physiological range, ultimately results from dysregulation of sodium reabsorption, water imbalance, or drug effects (e.g., hypertonic saline, desmopressin, and tolvaptan) [30]. Dysnatraemia is categorised into two clinical groups based on measured serum sodium concentration: hyponatraemia and hypernatraemia, defined most commonly by serum sodium concentration <135 mmol/L or >145 mmol/L, respectively [13]. Mechanisms for dysnatraemia onset can include a combination of neuroendocrine dysfunction in the setting of severe critical illness, primary CNS injury (i.e., TBI or SAH), chronic comorbidities, renal impairment, or drug effects [30,31]. Causes of acute hyponatraemia and hypernatraemia commonly identified in the ICU setting are summarised in Table 1.

Hyponatraemia causes include the syndrome of inappropriate antidiuretic hormone secretion (SIADH), cerebral salt wasting syndrome (CSW), Addison’s disease, severe hypothyroidism, overzealous treatment with desmopressin, a synthetic AVP analogue, and prolonged infusion of hypotonic fluid [32]. Amongst the critically ill population, hyponatraemia is particularly common after forms of brain injuries; it is seen in 50% of patients with SAH, 20% of patients with TBI, and 15% of those with brain tumours [32]. Regarding determinants of hyponatraemia at time of admission to ICU, it is more frequently observed in patients at extremities of age, surgical diagnosis, renal impairment, thyroid dysfunction, adrenal impairment, congestive cardiac failure, cirrhosis, and neurological or respiratory diagnoses [33].

Hypernatraemia is caused by inadequate free water intake, excess renal or extra-renal water loss, and rarely from excessive salt intake [1,13]. Amongst critically ill patients managed in the ICU setting, the most common causes are central or nephrogenic diabetes insipidus, hypercalcaemia, hypokalaemia, and diuretic use [1,32]. Some cases of hypernatraemia in critical illness are due to a patient’s lack of access to water combined with an impaired thirst response, meaning the occurrence of hypernatremia can reflect quality of ICU care [1,34]. Other cases of hypernatraemia are due to intentional use of sodium-based hyperosmolar fluid therapy, such as hypertonic saline, to reduce cellular swelling, particularly in the setting of primary CNS injury and critically raised intracranial pressure [35,36,37].

Prevalence of dysnatraemia in critically ill patients at time of admission to the ICU has been reported to be in up to one-third of cases [38,39,40], while up to another reported one-third of cases develop dysnatraemia during their ICU stay [41,42]. However, interpreting the prevalence data of dysnatraemia can be limited by population heterogeneity and differing serum sodium concentration cut-off definitions used in the literature [38,39,43]. Funk et al. defined borderline, mild, and severe hyponatremia as serum sodium concentration 130–135 mmol/L, 125–129, and <125, respectively [38], whereas Waikar et al. defined mild, moderate, and severe hyponatraemia as serum sodium concentration 130–134 mmol/L, 120–130, and <120, respectively [44]. Hypernatraemia has been defined as serum sodium concentration >145 mmol/L [45] or >150 mmol/L [46,47]; Aiyagari et al. defined mild, moderate, and severe hypernatraemia as serum sodium concentration 151–155 mmol/L, 156–160, and >160, respectively [46].

### 4.2. Physiological Effects

Sodium loss, if left uncorrected, causes ECF volume contraction (hypovolaemia) and increased sodium intake or reabsorption leads to ECF volume expansion (hypervolaemia), both of which can subsequently impact end-organ perfusion [18,19]. The organ most impacted by serum sodium concentration derangement is the brain and changing ECF osmolality also impacts brain volume and neuronal function [13].

Hyponatraemia is most often asymptomatic when mild, but cases of moderate to severe hyponatraemia, particularly in the acute setting, result in rapid widening of the osmotic gradient across the blood–brain barrier, promoting an intracellular shift of free water, leading to cerebral oedema, reduced cerebral oxygenation, brain herniation, risk of seizures, obtundation, and death [3]. Thus, avoiding hyponatraemia and the consequent deleterious secondary brain injury effects in the setting of primary CNS injury is paramount.

Hypernatraemia leads to neuronal dehydration, which is beneficial in the setting of globally swollen and injured brain, but in some cases this can cause consequent rupture of cerebral veins and resultant bleeding into the subarachnoid space [13]. Hypernatraemia is also associated with increased peripheral insulin resistance, impaired hepatic lactate clearance, decreased left ventricular cardiac contractility, and various clinical manifestations of neuromuscular dysfunction [1,48]. Symptoms of hypernatraemia can include thirst, lethargy, restlessness, muscle weakness, obtundation, and death [32]. Further, dynamic changes in serum sodium implies shifts in body water between fluid compartments and can have resulting consequences on haemodynamic regulation, neuron function, and impact secondary brain injury [26].

### 4.3. Clinical Outcomes

Due both to the pivotal role that the CNS plays in regulating serum sodium concentrations and to the deleterious effects of ICU-acquired dysnatraemia on brain tissue oxygenation and neuronal function, extensive literature on dysnatraemia has focused on brain-injured ICU populations [2,3,4,5,6,7,8,9]. Authors have argued that dysnatraemia and its complications can be minimised with early recognition and a systematic approach to diagnosis, but its workup and management has been shown to be inadequate on occasion [31,49]. A previous multi-centre study into hyponatraemia found that only 18% of general hospital patient populations underwent complete workup, and in many cases, the management plan was suboptimal [31]. A similar study focused on the ICU patient population would be invaluable due to the ICU being a limited resource and the report of ICU sodium correction being associated with a direct survival benefit [48].

Both categories of dysnatraemia reported amongst critically ill patients has been correlated with higher critical illness categorisation scores (i.e., Acute Physiology And Chronic Health Evaluation III (APACHE III)), longer ICU length of stay, higher mortality rates, and greater neurological morbidity [48,50,51,52,53]. Dysnatraemia present on admission to the ICU are commonly encountered in critically ill patients and have previously been associated with increased ICU mortality in mixed medical–surgical ICU cohorts upon adjustment for confounders [38]. As expected, the association between serum sodium concentration and mortality has been found to follow a U shape, with greater severity of hyponatraemia and hypernatraemia being associated with higher mortality [5,38]. The current literature is underpowered to determine whether dysnatraemia association with mortality is directly related or whether it is just a surrogate marker of the severe underlying disease process needing osmolality-changing drugs [48].

Although the APACHE III prognostic system has been validated to be a predictor of hospital mortality, there is little data to suggest whether dysnatraemia on admission is an independent predictor of mortality in all subgroups of neurological patients admitted to the ICU independent of illness severity prognostic systems [52]. Further, there is limited data that separates dysnatraemia on admission with dysnatraemia acquired in the ICU with regards to mortality or morbidity prognosis [52].

## 5. Ongoing Challenges

### 5.1. Sodium Measurement Techniques

An ongoing challenge to dysnatraemia in both clinical practice and research is a lack of standardisation to the method of serum sodium concentration measurement [54]. General hospital populations are likely to undergo ‘formal’ laboratory sample testing with flame photometry, a technique utilising an indirect ion-sensitive electrode (ISE), impacted by serum solid-phase composition [54]. This implies that plasma proteins, lipids, certain drugs, and metabolites can lead to spurious readings consistent with pseudohyponatraemia and pseudohypernatraemia [55,56]. In contrast, ICUs frequently use point-of-care analysers for arterial blood gas and electrolyte concentration measurement, a technique utilising a direct ISE and unaffected by the serum solid-phase composition [55,56]. This is also the technique considered the safer option for both initial classification of serum sodium status and during subsequent dysnatraemia correction [54].

Inter-method differences of at least |4| mmol/L or greater have been reported in ICU populations, which has profound impacts on clinical decision-making and also impacts patient data capturing in the context of research [54]. Direct ISEs are not universally available in all ICUs and interpreting treatment response from a mixture of indirect and direct ISE methods in the same patient can be problematic but may be commonplace in resource-limited ICUs. Furthermore, because direct and indirect ISE methods are not considered interchangeable, taking into consideration how patients with dysnatraemia are identified in ICU population studies is crucial.

### 5.2. Diagnostic Workup

The practical approach to dysnatraemia includes treatment of the underlying disease as well as restoring the distorted water and sodium balances. In the setting of critical illness, investigating for underlying disease is particularly challenged by the combined effects of (a) difficulty accurately measuring ECF volume status, (b) prolonged use of normal saline therapy, (c) drugs effect, and (d) multiple pathological processes [1,34,57]. ECF volume and total body sodium are estimated by a thorough history, physical examination and measurement of serum sodium concentration, and fractional urinary sodium excretion [58]. However, this approach may prove difficult in patients undergoing positive pressure ventilation, injured with severe burns or critically raised intracranial pressure, where a thorough history, physical examination of ECF volume status, and treatment responses may be difficult to elucidate [3,59].

Although AVP has been established as a key hormone involved in regulating serum sodium concentration (Figure 1), measuring serum AVP for diagnosing dysnatraemia aetiology has proven to be difficult due to its ultra-short half-life and tendency to bind to platelets [60,61,62]. As such, copeptin, a more stable C-terminal peptide cleaved off in a one-to-one ratio from pro-AVP, the precursor molecule of AVP, has been repeatedly used as a surrogate marker for AVP [62,63,64]. Amongst critically ill patients with hyponatraemia that prove difficult to measure volume status, using copeptin has been useful in cases, but stronger prospective data is warranted [62,63,64]. In the case of hyponatraemia from CSW, authors have implicated a potential role of ANP and BNP in the pathogenesis [12,23,24,25]. However, the studies into ANP and BNP in hyponatraemia and SAH, where CSW is encountered most frequently, have been small and underpowered and further, BNP is thought to be an unreliable predictor of ECF volume status in SAH [23,24,25,65].

Normal saline is the most commonly prescribed, often judiciously, crystalloid fluid therapy in the ICU and can cause profound changes to serum and urine sodium and osmolality and can impact dysnatraemia diagnosis workup [66]. Further, amongst critically ill patients with high AVP and natriuretic peptide levels, administering normal saline generates a ‘desalination phenomenon’, which can worsen hyponatremia secondary to natriuresis, with the renal production of sodium-free water [67]. Administration of desmopressin for enuresis or von Willebrand disease can cause severe cases of ICU-acquired hyponatremia [68]. Discontinuing desmopressin for correcting hyponatremia can cause brisk water diuresis, overly rapid correction of serum sodium level, and osmotic demyelination [68].

Contrastingly, hypernatremia is considered primarily a water problem rather than sodium excretion problem, as it usually results from inaccessibility to water, free water diuresis, or when there is a disordered thirst response [1,34]. Hypernatraemia can also occur amongst the critically ill secondary to reduced posterior pituitary release of AVP, the underlying pathological process in central diabetes insipidus, typically post severe TBI or post-pituitary surgery [69]. In rare cases, patients can undergo an inadequate renal response to AVP from auto-immune degradation of aquaporin-2 channels, called nephrogenic diabetes insipidus [70,71]. Identifying these patients, who will have normal AVP or copeptin, is crucial, as they do not respond to desmopressin, free water, or 5% dextrose, but rather require cessation of the causative drug, correction of hypercalcaemia and hypokalaemia, along with low sodium diet and thiazide and amiloride augmented-diuresis [70,71,72].

### 5.3. Tailoring Management

There is also disparity in the literature regarding the optimal management approach to dysnatraemia in the ICU, although authors argue that it should be largely tailored to the patient based on severity, chronicity, underlying cause, and its reversibility [51]. For hyponatraemia, treatments range from fluid restriction, urea, demeclocycline, loop diuretics, hypertonic saline, and AVP receptor antagonists (e.g., conivaptan and tolvaptan), each with specific associated benefits and drawbacks [73]. Hypertonic saline has received the most interest in the literature over recent years, but due to the requirement of reliable large-bore central venous access, risk of rapid serum sodium over-correction, and lack of consensus on the optimal dosing protocol or end sodium concentration target, and it is not so universally embraced in daily practice [49,74].

In the neurological ICU setting, hyponatraemia secondary to CSW and SIADH frequently occurs amongst patients with SAH and some authors argue differentiating between the two diagnoses is not essential and both can be corrected with hypertonic saline [75,76]. Given CSW is thought to be driven by a CNS-driven natriuretic peptide release and renal natriuresis, vigorous replenishment of intravenous sodium load with hypertonic saline is appropriate [76], but SIADH is secondary to renal reabsorption of free water and fluid restriction is typically considered the cornerstone of treatment [76]. However, fluid restriction amongst SAH patients leads to significantly increased risk of symptomatic vasospasm, but alternatively, routine implementation of hypertonic saline in undifferentiated patients with ICU-acquired hyponatraemia is not risk-free either [49,74].

There is limited, robust randomised controlled trial (RCT)-level evidence for the management of dysnatraemia and most intervention recommendations are based on expert consensus [77,78,79]. The majority of RCTs found evaluated AVP receptor antagonists for hyponatraemia and this emphasises paucity in data for the interventions such as desmopressin, hypertonic saline, and fludrocortisone that are anecdotally considered the “standard of care” [80,81,82]. One major criticism of existing AVP receptor antagonist-related RCTs for the management of hyponatraemia is the considerable publication bias because they are mostly industry-sponsored [80,81,82]. Further, there are limited RCTs to compare with other hyponatremia-related interventions. The only RCT for the use of 3% hypertonic saline in the management of hyponatraemia is the SALSA study, which found no difference in risk of sodium overcorrection between rapid intermittent bolus and slow continuous infusion regimens [83]. One RCT for the use of hydrocortisone vs. placebo for preventing hyponatraemia amongst patients with SAH was uncovered, and although hydrocortisone caused less hyponatraemia, there was no difference in symptomatic vasospasm [84]. It should also be emphasised that all RCTs comparing AVP receptor antagonists to placebo or fluid restriction used the surrogate endpoint of changes in serum sodium concentration and had no patient-centred primary outcome (e.g., ICU mortality) [80,81,82]. Extrapolating clinical impacts from changes in serum sodium of 2–5 mmol/L reported in many of the AVP receptor antagonist studies is challenging [80,81,82]. Currently, the evidence from clinical trials for AVP receptor antagonists suggests that they are safe with a low likelihood of adverse events; however, the lack of patient-focused outcomes suggests that they should not yet be widely used until more robust evidence. Furthermore, given the consideration of financial constraints in many ICUs, the cost associated with the use of AVP receptor antagonists, compared to current standard therapies, may be a barrier to widespread implementation.

### 5.4. Rate of Correcting Serum Sodium Concentration

Regarding rate of sodium concentration correction in dysnatraemia, the literature varies between maximum rates of 8–12 mmol/L over 24 h for acute hyponatraemia and hypernatraemia [69,72,77,78]. Rapid overcorrection of hyponatraemia through sodium loading or free water excretion theoretically risks osmotic central pontine demyelination and the debilitating ‘locked-in syndrome’ [75,77,78]. As such, avoiding overcorrection of acute hyponatraemia with hourly monitoring of serum sodium has been proposed, but it is only practical in resource-abled ICUs with frequent blood sampling from arterial lines [85]. In contrast, rapid overcorrection of hypernatraemia, a condition driven by free water deficit, is extremely unlikely and still probably safe, particularly in the older ICU patient, as cerebral atrophy compensates for reducing intracranial space if cerebral oedema begins to develops [69].

### 5.5. Gaps in Research

Several pertinent and clinically relevant aspects around ICU dysnatraemia remain unanswered. Firstly, the optimal serum sodium target level remains a topic of contention and whether different serum sodium targets for conditions such as TBI or SAH are needed is unclear. Secondly, there is growing literature support that dynamic changes in serum sodium amongst various heterogenous hospital populations has higher association with mortality as well as mortality and delayed cerebral ischaemia amongst patients with SAH [5,7,8,9,10]. Further research attention is needed on whether clinicians should consider lower thresholds when correcting dysnatraemia in the ICU based on the notion that even small variations of serum sodium levels may be detrimental.

The bulk of existing literature on serum sodium has been observational, and the reported associations amongst epidemiological studies may simply be reflective of the underlying primary disease severity or treatment response. Again, given that only a handful of dysnatraemia studies have been at the RCT level, one’s capacity to make strong recommendations for daily ICU practice is somewhat limited. However, the overall impression from the literature is that dysnatraemia encountered in the ICU is a heterogenous process and varied ICU resource capacities worldwide must be considered, so a standardised regimen may never be established.

Another consideration is that epidemiological studies that reported incident dysnatraemia in the ICU as a static parameter and associate it with patient outcomes may be of limited value. In future research, serum sodium needs consideration as a dynamic parameter where magnitude and duration (i.e., dosage of exposure to dysnatraemia) are rather studied and associated to clinical outcomes.

## 6. Conclusions

Dysnatraemia is commonly encountered amongst critically ill patients in the ICU setting and associated with higher mortality and morbidity. However, it remains unclear whether the dysnatraemia is a marker of inherent poor prognosis from the primary disease or an active contributor to adverse outcomes, but the evidence is mixed. Further, the critically ill population managed in the ICU are a separate entity to the general hospital population and should be taken into consideration during the diagnosis and management process. A practical approach to these patients requires a comprehensive understanding of sodium and water balance regulation but also recognising the nuanced challenges encountered in the ICU compared to the general hospital setting.

## Figures and Tables

**Table 1:** Common causes of acute hyponatraemia and hypernatraemia in the ICU setting.

Hyponatraemia	Hypernatraemia
Hypertonic (>285 mOsm/L) Hyperglycaemia Mannitol * Isotonic (275–285 mOsm/L) Hyperproteinaemia Hyperlipidaemia Hypotonic (<275 mOsm/L) SIADH CSW Addison’s Disease Diuretics Desmopressin Hypotonic Fluid Therapy Acute Kidney Injury Severe Hypothyroidism Severe Vomiting Severe Diarrhoea	High Urine Volume (>3 L/24 h) Diabetes Insipidus Hypertonic Saline Osmotic Diuresis (e.g., Mannitol) Low Urine Volume (<3 L/24 h) Low level of consciousness/no access to water Severe Burns

* Note: Mannitol can cause both hyponatraemia and hypernatraemia.

## Abbreviations

ANP = atrial natriuretic peptide, APACHE III = Acute Physiology And Chronic Health Evaluation III, AVP = arginine vasopressin, BNP = brain natriuretic peptide, CNS = central nervous system, CSW = cerebral salt wasting, ECF = extracellular fluid, ICU = intensive care unit, RAAS = renal–angiotensin–aldosterone system, RCT = randomised controlled trial, SAH = subarachnoid haemorrhage, SIADH = syndrome of inappropriate antidiuretic hormone secretion, TBI = traumatic brain injury.
